# Use of Virtual Reality in the Reduction of Pain After the Administration of Vaccines Among Children in Primary Care Centers: Protocol for a Randomized Clinical Trial

**DOI:** 10.2196/35910

**Published:** 2022-04-07

**Authors:** Mercedes de la Cruz Herrera, Aïna Fuster-Casanovas, Queralt Miró Catalina, Mireia Cigarrán Mensa, Pablo Alcántara Pinillos, Isabel Vilanova Guitart, Sergi Grau Carrión, Josep Vidal-Alaball

**Affiliations:** 1 Centre d'Atenció Primària Súria Gerència Territorial de la Catalunya Central Institut Català de la Salut Sant Fruitós de Bages Spain; 2 Gerència Territorial de la Catalunya Central Institut Català de la Salut Sant Fruitós de Bages Spain; 3 VRPharma Immersive Technologies SL Barcelona Spain; 4 Digital Care Research Group Centre for Health and Social Care Research University of Vic-Central University of Catalonia Vic Spain; 5 Health Promotion in Rural Areas Research Group Gerència Territorial de la Catalunya Central Institut Català de la Salut Sant Fruitós de Bages Spain; 6 Unitat de Suport a la Recerca de la Catalunya Central Fundació Institut Universitari per a la Recerca a l'Atenció Primària de Salut Jordi Gol i Gurina Sant Fruitós de Bages Spain; 7 Faculty of Medicine University of Vic-Central University of Catalonia Vic Spain

**Keywords:** children, virtual reality, pain, pain perception, anxiety, vaccination, immunization schedule

## Abstract

**Background:**

Pain and anxiety caused by vaccination and other medical procedures in childhood can result in discomfort for both patients and their parents. Virtual reality (VR) is a technology that is capable of entertaining and distracting the user. Among its many applications, we find the improvement of pain management and the reduction of anxiety in patients undergoing medical interventions.

**Objective:**

We aim to publish the protocol of a clinical trial for the reduction of pain and anxiety after the administration of 2 vaccines in children aged 3 to 6 years.

**Methods:**

We will conduct a randomized, parallel, controlled clinical trial with 2 assigned groups. The intervention group will wear VR goggles during the administration of 2 vaccines, while the control group will receive standard care from a primary care center for the procedure. Randomization will be carried out by using the RandomizedR computer system—a randomization tool of the R Studio program. This trial will be an open or unblinded trial; both the subjects and the investigators will know the assigned treatment groups. Due to the nature of the VR intervention, it will be impossible to blind the patients, caregivers, or observers. However, a blind third-party assessment will be carried out. The study population will include children aged 3 to 6 years who are included in the patient registry and cared for in a primary care center of the region of Central Catalonia. They will receive the following vaccines during the Well-Child checkup: the triple viral+varicella vaccine at 3 years of age and the hepatitis A+diphtheria-tetanus-pertussis vaccine at 6 years of age.

**Results:**

The study is scheduled to begin in January 2022 and is scheduled to end in January 2023, which is when the statistical analysis will begin. As of March 2022, a total of 23 children have been recruited, of which 13 have used VR during the vaccination process. In addition, all of the guardians have found that VR helps to reduce pain during vaccination.

**Conclusions:**

VR can be a useful tool in pediatric procedures that generate pain and anxiety.

**International Registered Report Identifier (IRRID):**

PRR1-10.2196/35910

## Introduction

Pain is defined as a complex and multidimensional sensory experience that comprises cognitive, behavioral, and psychological elements [1,2]; it is usually associated with unpleasant and subjective experiences and involves an adaptive function that allows for the initiation of protective responses [2]. Fear and stress influence the perception of pain, and the failure to apply appropriate pain reduction techniques exposes patients to unnecessary negative experiences, which can lead to long-term consequences, such as needle phobia or anxiety about medical procedures [2-4].

Pediatric patients seen in primary care settings frequently experience negative reactions, such as fear, anxiety, pain, or feelings of aggression, during routine invasive procedures, such as vaccinations, blood draws, wound sutures, or nursing care.

According to the World Health Organization, the possible adverse reactions to vaccination that are linked to procedural stress include fainting, hyperventilation, vomiting, and seizures [5]. Further, 19% of patients aged 4 to 6 years have needle phobia [6], with the average age of onset being 5.5 years [7]. Additionally, 7% of children experience syncope (fainting) during the administration of a medical injection, and 5% avoid treatment [8]. In a study on the impact of the fear of needles on adherence to vaccination, it was estimated that fear was the cause of nonadherence in 8% of children [9].

For all of these reasons, different mechanisms have been developed to try to minimize or eliminate adverse reactions to vaccination [2]. In the clinical practice guidelines for reducing pain during the administration of vaccines in the pediatric age group [10,11], the following main recommendations are made: breastfeeding during the procedure, offering sweet solutions before vaccine administration to those older than 1 year if they cannot be breastfed, and comfortably positioning patients if they can be held by their parents or using a rapid vaccination technique without aspiration.

Distractions help to reduce anxiety because they prevent painful stimuli from being transmitted either to the thalamus (ie, to the limbic system) or to the sensory cortex in such an effective way [12,13], thereby helping one to focus their attention on external and internal stimuli and not on nociceptive stimuli [14,15]. Distractions sometimes surpass local anesthetics’ ability to control pain and discomfort associated with medical interventions [12]. Distractions can be active (immersive), which involves patient participation through the manipulation of the environment, or passive (nonimmersive), which involves observation only [13].

Current technological advances, especially in the field of virtual reality (VR), have resulted in new types of distractions, which can be used along with traditional distractions [14] to achieve better pain control in pediatric patients for procedures such as vaccination, according to the *Protocol of Preventive and Health Promotion Activities in Pediatric Patients: Healthy Childhood* [15].

*VR* is a term that was proposed in the mid-1980s by Jaron Launier, and it refers to a computer technique that allows for the creation of a simulated environment by means of a device with sensors that can be connected to a computer, mobile device, or tablet [1,3]. Its effectiveness is based on the psychological theory of “presence” [2,3]. This theory posits that people interact with their environments via the following three types of components: auditory, visual, and tactile components [1,2]. VR technology redirects one’s attention to a more pleasant environment [2] by replacing real stimuli with virtual stimuli. This activates users’ higher cognitive and emotional brain regions, resulting in the dissociation of pain [2,3].

The idea behind VR-generated analgesia was probably inspired by the intercortical modulation of pain matrix signaling pathways via attention, emotion, memory, and other senses [12,13].

In a systematic review published by the Cochrane Library in 2019 on VR distraction for reducing acute pain in children, it was concluded that the use of VR had low evidence regarding its benefits. However, in that review, due to the limited amount of data available, no conclusions could be drawn about the side effects of VR, satisfaction with VR, the level of parental anxiety, or the cost of VR use [12].

Many aspects remain to be clarified; however, the use of VR for pain management has shown great benefit in hospital procedures. Our study seeks to introduce VR as an analgesic tool in pediatric primary care services and daily procedures, such as vaccination, thereby combining new technologies with traditional concepts, such as distraction [1-3].

## Methods

### Study Design

#### Trial Design

We will conduct a randomized, single-center, open, parallel, and controlled clinical trial with 2 assigned groups (intervention group and control group).

#### Scope and Period of the Study

The study population will include children aged 3 to 6 years who are included in the patient registry and are being seen in a primary care center of the Catalan Institute of Health in Central Catalonia.

The study will be conducted during the period of January 2022 to January 2023. If the minimum sample size is not achieved, the study period will be extended until it is achieved.

#### Participants

##### Inclusion Criteria

The patients who will take part in the study will be those from the pediatric population (ie, those aged 3 to 6 years) in the register of patients from a primary care center of the Catalan Institute of Health in Central Catalonia who, according to the vaccination schedule, are due to receive 1 of the following 2 vaccinations: (1) the triple viral+varicella vaccine at 3 years of age and (2) the hepatitis A+diphtheria-tetanus-pertussis vaccine at age 6.

##### Exclusion Criteria

The exclusion criteria will include the following: (1) patients who have already received 1 of the 2 vaccines to be administered; (2) patients and accompanying persons who do not understand and speak Catalan or Spanish; (3) patients with physical or mental illnesses, as well as those with blindness or deafness; (4) patients with a known history of epileptic episodes or severe motion sickness; (5) patients with any infections, burns, or injuries to the face, head, or neck that may interfere with the placement of the VR device; and (6) the absence of legal guardians for signing the informed consent form.

#### Intervention

The intervention group will use the Pico G2 VR goggles (Pico Interactive Inc) during the administration of the two vaccines, together with an Android AOYODKG tablet, which will be connected to the goggles as a controller.

The control group participants will receive traditional distractors, such as being held by the parent or guardian who accompanies them to the appointment, receiving stickers at the end of the appointment, or receiving rewards that the parent or guardian has prepared from home.

Eligible patients will be invited to participate in the study on an ongoing basis, and the assignment to study groups will be randomized.

#### Randomization

##### Sequence Generation

Randomization will be carried out by using the RandomizedR computer system—a randomization tool of the R Studio program.

##### Implementation

The participants will be selected from a patient diary register. Both the sequence and the allocation of participants to the interventions will be generated by using the RandomizedR computer system.

##### Masking

Due to the nature of the study, it will not be possible to mask patients or health care professionals. Therefore, the trial will be open or unblinded. However, a blind evaluation by third parties will be carried out, as the person in charge of data analysis will not be involved with the intervention.

#### Sample Size Determination

To detect a 1-point difference between the two groups on the pain level scale, a sample of 150 boys and girls in each group is required, assuming an SD of 3 points, an α risk of 5%, a power of 80%, and an estimated loss to follow-up rate of 5% [16].

### Data Collection and Sources of Information

#### Recruiting Patients to Participate in the Study

The procedure will be carried out by 1 pediatric team consisting of a pediatrician and a nurse of the primary care center in Súria (Spain). The families will be contacted by telephone to schedule the patients for the checkup at 3 and 6 years of age, as is currently done. The nurse will administer the vaccines and will continue to be part of the Well-Child checkup team.

Information on the purpose, risks, and benefits of the study will be provided, and any queries will be answered. In addition to verbal information, an information document about the study will be provided.

Prior to the start of the study, training on the use of the devices will be given to all of the health care personnel involved.

If a family agrees to be included in the study, the informed consent form must be signed by at least 1 of the parents or legal guardians. The signing parent or guardian will agree to inform the other parent or guardian.

#### Data Collection, Sources of Information, and Intervention

Study data collection will begin once informed written consent has been obtained from the legal guardian. For patients’ assignment to a study group, randomization will be carried out by using the RandomizedR computer system. The person responsible for data collection will indicate the patients’ age, gender, and study group and the type of intervention performed. Prior to administering the vaccine, the patients’ condition and heart rate on arrival at the clinic will be recorded, regardless of their assigned study group.

For patients in the intervention group, it will be explained to them that they will be able to use the VR device; they will be assisted in fitting the device and will be given a brief explanation of the content that will be played. The *Leia's World* (VRPharma Immersive Technologies SL) content, which was specially designed for vaccination, will be used, and data will be collected before and after the procedure for each of the first 2 vaccinations.

The Wong-Baker Faces Pain Rating Scale, which ranges from 0 to 10, and the Children’s Fear Scale, which ranges from 0 to 4, will be used to evaluate the reduction of pain and anxiety. The data collected from the control group will be compared with those collected from the intervention group (heart rate, the level of pain perception, the level of distress and fear, and the length of visits) [17-19]. In addition, to understand the perceptions of the tutors on the use of VR, a satisfaction survey will be conducted [20].

The aforementioned data will be collected by the nursing or pediatric professional via a web questionnaire generated by the Microsoft Forms tool on the tablet and will be hosted in a computer server of the Institut Català de la Salut de la Catalunya Central.

### Statistical Analysis

An intention-to-treat analysis will be performed; the subjects will be analyzed according to the group to which they were initially assigned and not the group in which they finally participated.

The data will be obtained through Microsoft Forms (an application included in Office 365 [Microsoft Corporation] that allows one to create customized questionnaires, surveys, and records) and analyzed with R software (version 4.0.3) [21,22]. Categorical variables will be described with absolute frequencies and percentages, and continuous variables will be described with means and SDs or medians and quartiles. A 2-tailed *t* test will be used to compare the values related to pain, anxiety, and satisfaction across the two groups. The correlations between pain perception and anxiety values reported by the children and those reported by their caregivers and nurses will be evaluated by means of a Pearson correlation. The significance level will be set at 5%, and all CIs will be set at 95%.

The data will be stored in a database. The Pearson chi-square test will be used for the calculation of statistical significance.

### Ethics Approval

The University Institute for Research in Primary Health Care Jordi Gol i Gurina (Barcelona, Spain) ethics committee approved the trial study protocol (approval code: 21/233). Written informed consent will be requested from all parents or legal guardians participating in the study.

## Results

The study is scheduled to begin in January 2022 and is scheduled to end in January 2023, which is when the statistical analysis will begin. As of March 2022, a total of 23 children have been recruited, of which 13 have used VR during the vaccination process. In addition, all of the guardians have found that VR helps to reduce pain during vaccination.

We hope that sufficient evidence can be obtained to demonstrate that the use of VR is effective in reducing anxiety and pain. In this context, the Catalan health system could introduce the use of VR in usual practices and extend its use to other potentially painful processes. Statistically significant differences in heart rate and decreased pain perception that are in favor of the intervention group will be considered a satisfactory result.

## Discussion

Our study aims to demonstrate that the use of VR goggles reduces the pain reported by children aged 3 to 6 years during the administration of 2 vaccines. The use of VR goggles may also reduce anxiety after such a procedure and thus result in greater satisfaction among the parents or legal guardians.

There are studies that have already used VR with children for painful procedures and during the administration of vaccines [12-14]. However, to date, there is no literature describing studies that focus on the population of children aged 3 to 6 years who are administered 2 vaccines at the same visit and also record the adverse effects of the use of VR goggles. In this context, our study may provide further support for the use of VR in the management of pediatric patients. Obtaining favorable results could lead to the use of VR as a standard practice for painful procedures performed in the primary care centers of Catalonia. The use of VR for pain reduction is likely to result in a decrease in visit duration. To demonstrate the efficacy of the use of VR, the professionals will note the length (in minutes) of the visits in order to evaluate whether VR reduces or increases the duration of the visits.

Our study has several limitations. The main limitation of the study is the pressure of care and time management in the pediatric consultations conducted by the Well-Child Programme. The time available for caring for a patient is limited. The time required for secluding the participants and obtaining consent and questionnaire data is estimated to be 5 minutes. This means that if the burden of care is high, the quality of care will have to be prioritized, and the recruitment of patients who are eligible to participate in the study will have to be paused.

Another limitation is that parents (or legal guardians) and minors who do not speak Catalan or Spanish cannot participate in the study. The use of VR could be highly advantageous if the VR content is translated into the native languages of these children, because they are usually more fearful and anxious when they cannot understand the pediatrician or nurse.

There are other limitations associated with the study design, patient recruitment, and the inclusion and exclusion criteria. First, patients receiving vaccines from their private pediatricians will be excluded. Second, the study population will be limited to a specific age range. Third, evaluations will only be conducted for vaccination and not for other invasive procedures. Fourth, patients who have already received 1 of the 2 scheduled vaccines will be excluded, since they would have previously presented the related pathology (eg, patients who have already been vaccinated for chickenpox). Fifth, parents or legal guardians who do not agree with vaccination in general or are associated with the masking process may introduce bias when they report their experiences in the survey.

## Abbreviations

VR: virtual reality

## Data Availability

The principal study researchers will have access to the full data set, and the data generated and analyzed during the study will be available from the corresponding author. The results obtained are expected to be published in peer-reviewed journals and at national and international conferences.
